# *Populus euphratica* CPK21 Interacts with NF-YC3 to Enhance Cadmium Tolerance in Arabidopsis

**DOI:** 10.3390/ijms25137214

**Published:** 2024-06-29

**Authors:** Kexin Yin, Yi Liu, Zhe Liu, Rui Zhao, Ying Zhang, Caixia Yan, Ziyan Zhao, Bing Feng, Xiaomeng Zhang, Keyue An, Jing Li, Jian Liu, Kaiyue Dong, Jun Yao, Nan Zhao, Xiaoyang Zhou, Shaoliang Chen

**Affiliations:** 1 State Key Laboratory of Efficient Production of Forest Resources, College of Biological Science and Technology, Beijing Forestry University, Beijing 100083, China; ykx0303@126.com (K.Y.); ly4862ccc@163.com (Y.L.); liuz6415@163.com (Z.L.); ruizhao926@126.com (R.Z.); zying@bjfu.edu.cn (Y.Z.); caixiayan2019@163.com (C.Y.); zzyan913@163.com (Z.Z.); 18720795166@163.com (B.F.); zhangxiaomeng@bjfu.edu.cn (X.Z.); ankeyue@bjfu.edu.cn (K.A.); lijing70747@163.com (J.L.); liujian20170703@163.com (J.L.); 18353541623@163.com (K.D.); zhaonan19880921@126.com (N.Z.); zhouxiaoyang@bjfu.edu.cn (X.Z.); 2 Guangdong Provincial Key Laboratory of Silviculture, Protection and Utilization, Guangdong Academy of Forestry, Guangzhou 510520, China; yaojun990@126.com

**Keywords:** *Populus euphratica*, PeCPK21, AtNF-YC3, cadmium, Cd fluxes, H_2_O_2_, enzyme activities

## Abstract

The toxic metal cadmium (Cd) poses a serious threat to plant growth and human health. *Populus euphratica* calcium-dependent protein kinase 21 (CPK21) has previously been shown to attenuate Cd toxicity by reducing Cd accumulation, enhancing antioxidant defense and improving water balance in transgenic Arabidopsis. Here, we confirmed a protein–protein interaction between PeCPK21 and Arabidopsis nuclear transcription factor YC3 (AtNF-YC3) by yeast two-hybrid and bimolecular fluorescence complementation assays. *AtNF-YC3* was induced by Cd and strongly expressed in *PeCPK21*-overexpressed plants. Overexpression of *AtNF-YC3* in Arabidopsis reduced the Cd inhibition of root length, fresh weight and membrane stability under Cd stress conditions (100 µM, 7 d), suggesting that AtNF-YC3 appears to contribute to the improvement of Cd stress tolerance. AtNF-YC3 improved Cd tolerance by limiting Cd uptake and accumulation, activating antioxidant enzymes and reducing hydrogen peroxide (H_2_O_2_) production under Cd stress. We conclude that PeCPK21 interacts with AtNF-YC3 to limit Cd accumulation and enhance the reactive oxygen species (ROS) scavenging system and thereby positively regulate plant adaptation to Cd environments. This study highlights the interaction between PeCPK21 and AtNF-YC3 under Cd stress conditions, which can be utilized to improve Cd tolerance in higher plants.

## 1. Introduction

Contamination of soils with cadmium (Cd) disrupts plant growth and endangers human health [1,2]. Ca^2+^ signaling and Ca^2+^-dependent protein kinase (CPK) have been shown to be crucial for the adaptation of plants to Cd environments [3,4]. AtCPK21 and AtCPK23 interact with natural resistance-associated macrophage protein 6 (NRAMP6) and limit Cd transport in Arabidopsis [3]. Recently, the calcium sensor PeCPK21 from *Populus euphratica* was found to interact with heavy metal transport proteins, plant defensin-like protein 2.2 (PDF2.2), copper transporter 5 (COPT5), oligopeptide transporter 3 (OPT3) and annexin (ANN), and subunits of vacuolar ATPases, V-type proton ATPase proteolipid subunit (AVA-P2), V-type proton ATPase subunit B1 (VHA-B1) and V-type proton ATPase subunit C (VHA-C) to control Cd homeostasis [4]. Our previous studies have shown that *P. euphratica* attenuates Cd toxicity by limiting Cd absorption and increasing Cd compartmentalization [5,6]. It is noteworthy that *P. euphratica* decreases the expression of *ANN1* to limit Cd accumulation, as ANN1 promotes Cd entry through Ca^2+^-permeable channels (CaPCs) [7,8]. The addition of abscisic acid (ABA) leads to the activation of antioxidant enzymes that effectively scavenge H_2_O_2_ in Cd-exposed *P. euphratica* cells and thus contributes to the limitation of Cd entry through CaPCs [9]. The molecule hydrogen sulphide (H_2_S) promotes Cd efflux and facilitates vacuolar Cd sequestration in *P. euphratica* cells [5]. In addition, *P. euphratica* up-regulates the transcription of xyloglucan endotransglucosylase/hydrolase (*XTH*) and promotes xyloglucan degradation, which leads to a reduction in binding sites and thus reduces Cd accumulation in the roots [6]. To mitigate the damage caused by Cd stress, plants can also use non-enzymatic and enzymatic antioxidant defense systems to scavenge the Cd-triggered reactive oxygen species (ROS) [5,9]. Catalase (CAT), peroxidase (POD), ascorbate peroxidase (APX) and superoxide dismutase (SOD) are dominant enzymes in the plant defense strategies [10,11,12,13]. PeCPK21 has been shown to interact with chloroplastic drought-induced stress protein of 32 kDa (CDSP32), glutathione peroxidase (GPX3), ascorbate peroxidase 1 (APX1), ascorbate peroxidase 2 (APX2), thylakoid ascorbate peroxidase (TAPX), thioredoxin M4 (TRXM4) and thioredoxin superfamily protein (PRXQ) to maintain ROS homeostasis under Cd stress [4]. PeCPK21 regulates water status by interacting with intrinsic proteins in the plasma membrane, plasma membrane intrinsic protein 2A (PIP2A), plasma membrane intrinsic protein 1-1 (PIP1–1) and plasma membrane intrinsic protein 2-7 (PIP2–7) [4], as water transport is severely restricted in Cd-stressed roots [14]. Although PeCPK21 attenuates Cd stress by interacting with various heavy metal stress-associated proteins (HMAPs) in transgenic Arabidopsis [4], it is unknown whether PeCPK21 interacts with transcription factors to control Cd and ROS homeostasis in stressed plants.

The transcription factor (TF), nuclear factor Y (NF-Y) or heme-activated protein (HAP) consists of three different subunits, NF-YA, NF-YB and NF-YC [15]. NF-Y regulates crucial aspects of growth, development and environmental stress responses [16,17,18,19,20,21,22,23]. For example, *AsNF-YC8* from garlic positively regulates plant tolerance to hyperosmotic stress in tobacco [24]. AtNF-YA5 is critical for the induction of drought-responsive genes in Arabidopsis [25]. AtNF-YB1 and ZmNF-YB2 improved drought resistance by regulating stomatal conductance [26]. NF-YC from *Amaranthus hypochondriacus* increases ABA sensitivity and confers resistance to water deficits in Arabidopsis [27]. Soybean GmNF-YC14 activates the GmPYR1-mediated ABA signaling pathway to regulate drought tolerance [28]. *Physcomitrella patens* PpNF-YC1 activates the *PpLEA1* promoter to enhance drought/desiccation tolerance [29]. *ShNF-YC9* expression has been shown to be elevated in the leaf and root of *E. arundinaceus* under drought treatment [30]. *ZmNF-YC12* has been shown to be highly induced by drought and positively regulates drought resistance and recovery ability [31]. The *NF-Y* genes *PgNF-YB09*, *PgNF-YC02* and *PgNF-YC07-04* were induced by salinity in *Panax ginseng* [32]. The *OsNF-YC13* gene increases salt tolerance in rice plants [33]. Under salinity stress, *EcNF-YC2* showed increased expression levels in crop finger millet [34]. The NF-YA1-YB2-YC9 complex promotes the expression of the salt-responsive gene *MYB75,* leading to increased salt tolerance in Arabidopsis [35]. *MsNF-YC2* overexpression confers alkali tolerance in transgenic alfalfa cultivars [36]. Bermudagrass Cdt-NF-YC1 improves the ability of transgenic rice to tolerate drought and salt stress [37]. AtHAP5A has been shown to modulate freezing tolerance in Arabidopsis [38]. *CmNF-YC1/C2/C5/C7/C8* were up-regulated stably under cold stress [39]. However, the function of NF-Y transcription factors under Cd stress is still unclear and remains to be investigated.

In our previous studies, the HaloTag pull-down protocol was used to enrich PeCPK21-interacting proteins [4]. By analyzing the PeCPK21-interacting proteins with a mass spectrometry assay, we also found that PeCPK21 interacts with the transcription factor AtNF-YC3. In this study, we confirmed the interaction of PeCPK21 with AtNF-YC3 by yeast two-hybrid (Y2H) and bimolecular fluorescence complementation (BiFC) experiments. We found that Cd induced *AtNF-YC3* expression in *PeCPK21*-transformed Arabidopsis. *AtNF-YC3* was transferred into Arabidopsis to further determine whether the PeCPK21-interacting TF could enhance Cd tolerance. The overexpression of *AtNF-YC3* in Arabidopsis resulted in decreased Cd uptake and activated antioxidant enzymes, which reduced H_2_O_2_ accumulation and improved Cd stress tolerance. Thus, in Arabidopsis overexpressed with *PeCPK21*, PeCPK21 interacts with AtNF-YC3 to decrease Cd accumulation and strengthen the antioxidant system to reduce the Cd-triggered ROS. This discovery of the interaction between PeCPK21 and AtNF-YC3 can be utilized to improve Cd resistance in higher plants.

## 2. Results

### 2.1. Cd-Induced AtNF-YC3 Expression in PeCPK21-Transgenic Arabidopsis

**Figure 1:**
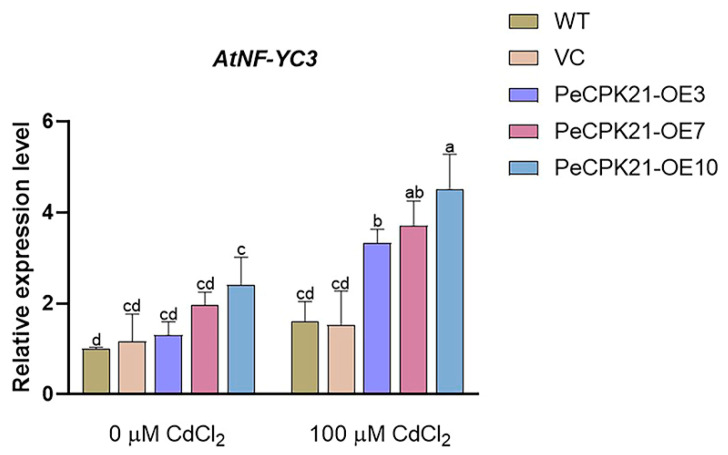
Cadmium (Cd)-induced transcription of *AtNF-YC3* in *PeCPK21*-transgenic Arabidopsis. Seedlings of wild-type (WT), vector control (VC) and *PeCPK21*-overexpressed (OE) lines OE3, OE7 and OE10 (T3 generation) were grown on 1/2 Murashige and Skoog (MS) medium supplemented with 0 or 100 μM CdCl_2_. RT-qPCR analysis of *AtNF-YC3* was performed after 7 days of Cd treatment. Data are mean values of three biological samples, and bars with different letters (a–d) indicate significant differences (*p* < 0.05).

We have previously shown that *P. euphratica* PeCPK21 enhances Cd tolerance in Arabidopsis, and the PeCPK21-interacting proteins were identified in *PeCPK21*-transgenic plants [4]. Expression profiles of PeCPK21-interacting proteins indicate that various HMAPs were up-regulated by Cd stress in transgenic Arabidopsis. In this work, we observed that the expression of the transcription factor AtNF-YC3 was up-regulated by Cd exposure in *PeCPK21*-overexpressed lines. The *AtNF-YC3* transcript increased significantly by 60–155% upon Cd exposure in the *PeCPK21*-overexpressed (OE) lines OE3, OE7 and OE10, which was 2.50-fold higher than in the wildtype (WT) and vector control (VC) (Figure 1). This result suggests that PeCPK21 may interact with AtNF-YC3 to increase Cd tolerance, as overexpression of *Picea wilsonii NF-YB3* in Arabidopsis increases Ca^2+^-dependent protein kinase 1 (*CDPK1*) expression and confers salt and drought tolerance [40]. Therefore, the interaction between PeCPK21 and AtNF-YC3 and the role of AtNF-YC3 in Cd tolerance were investigated in the present study.

### 2.2. AtNF-YC3 Sequence Analysis

**Figure 2:**
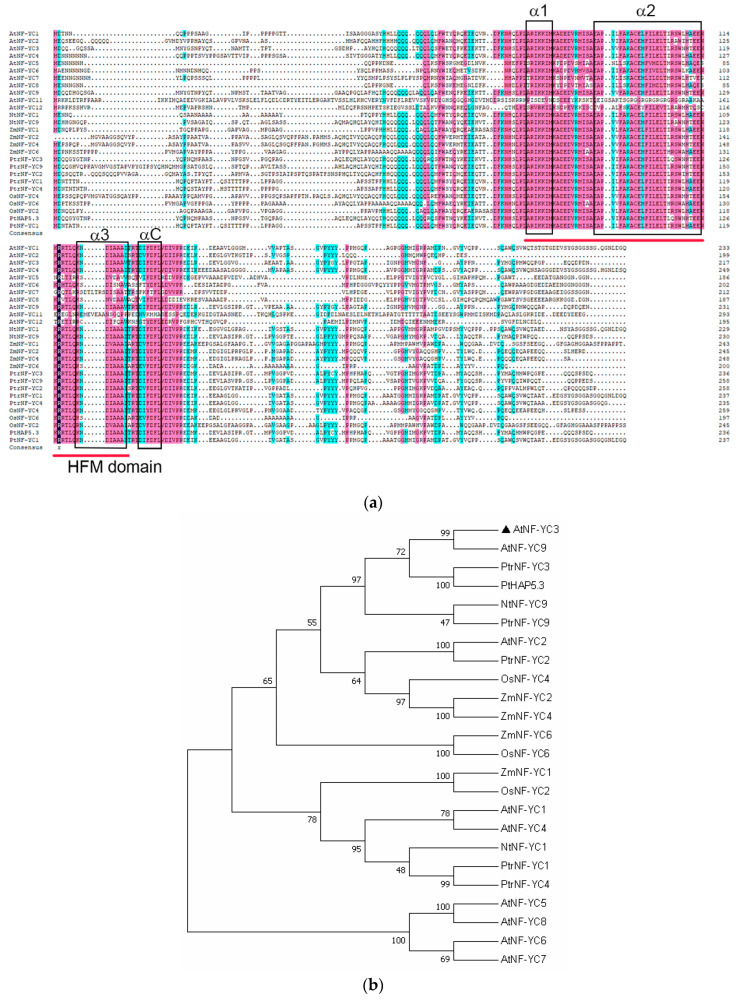
Sequence analysis of *Arabidopsis thaliana* nuclear transcription factor YC3 (AtNF-YC3). (**a**) Multiple sequence alignment of the NF-YC proteins. The blue and pink shadings indicate identical and conserved amino acid residues. The histone fold motif (HFM) domain is indicated by red lines, and the α-helices are shown by black boxes. (**b**) Phylogenetic analysis. The phylogenetic tree was constructed with the neighbor-joining method. At, *Arabidopsis thaliana*; Os, *Oryza sativa*; Nt, *Nicotiana tabacum*; Zm, *Zea mays*; Pt, *Populus tomentosa*; Ptr, *Populus trichocarpa*. Supplementary Table S3 lists the accession numbers of the NF-YC3 orthologues.

The coding sequence (CDS) of *AtNF-YC3* (654 bp) was isolated from *Arabidopsis thaliana*. AtNF-YC3 encodes 217 amino acids (24.32 kDa) with an isoelectric point of 4.75 (Figure 2a). The phylogenetic tree shows that AtNF-YC3 in *A. thaliana* has a close evolutionary relation to AtNF-YC9 (Figure 2b). The NF-YC conserved domain contains a histone fold motif (HFM) domain that plays an important role in protein–DNA and protein–protein interactions (Figure 2a). The domain consists of three α-helices (α1, α2 and α3) separated by two β-chain ring domains. Outside the HFM folding region is a fourth α-helix with a length of seven amino acids, called αC (Figure 2a).

### 2.3. Subcellular Localization of PeCPK21 and AtNF-YC3

**Figure 3:**
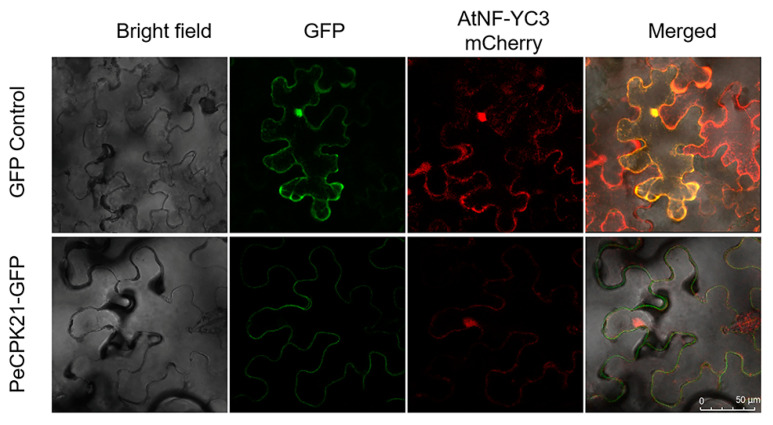
Subcellular localization of PeCPK21 and AtNF-YC3. *Agrobacterium tumefaciens* strains containing *PeCPK21*-GFP and *AtNF-YC3*-mCherry were injected together into tobacco leaves. GFP (Green) and mCherry (Red) fluorescence was observed under a confocal microscope (Leica SP8).

We determined the subcellular co-localization of PeCPK21 and AtNF-YC3 in leaves of *Nicotiana benthamiana*. Green fluorescent protein (GFP)-tagged PeCPK21 (PeCPK21-GFP), which was localized in the cytoplasm, was expressed together with mCherry-tagged AtNF-YC3 (AtNF-YC3-mCherry) in tobacco leaves, which was localized in the nucleus and cytoplasm (Figure 3). The colocalization assay showed that PeCPK21 and AtNF-YC3 exhibited overlapping fluorescence in the cytoplasm.

### 2.4. PeCPK21 Interacts with AtNF-YC3 In Vivo

In this study, yeast two-hybrid (Y2H) and bimolecular fluorescence complementation (BiFC) assays were performed to verify the interaction between PeCPK21 and AtNF-YC3.

**Figure 4:**
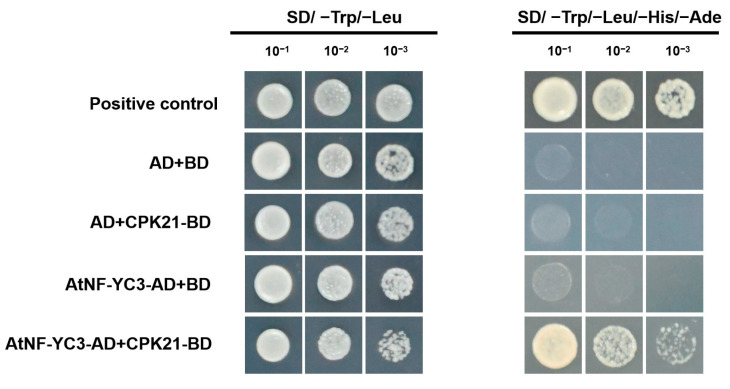
Yeast two-hybrid (Y2H) analysis between PeCPK21 and AtNF-YC3. The ratios 1:10, 1:100 and 1:1000 correspond to 10-, 100- and 1000-fold dilution, respectively. Yeast transformants were grown on synthetic dropout (SD) (-Leu/-Trp) control medium and SD (-Leu/-Trp/-His/-Ade) selection medium. AD, activating domain; BD, binding domain.

Y2H was used to investigate whether PeCPK21 interacts with AtNF-YC3 *in vivo*. Prior to transformation into yeast cells, AtNF-YC3 was linked to the GAL4 activation domain (AtNF-YC3-AD), and PeCPK21 was fused to the GAL4 DNA binding domain (PeCPK21-BD). The transformation combinations showed that only the AH109 yeast cells carrying AtNF-YC3-AD and PeCPK21-BD could grow on the selection medium (synthetic dropout (SD)/-Trp/-Leu/-His/-Ade). Therefore, the Y2H assays showed that PeCPK21 could interact with AtNF-YC3 in yeast cells (Figure 4).

**Figure 5:**
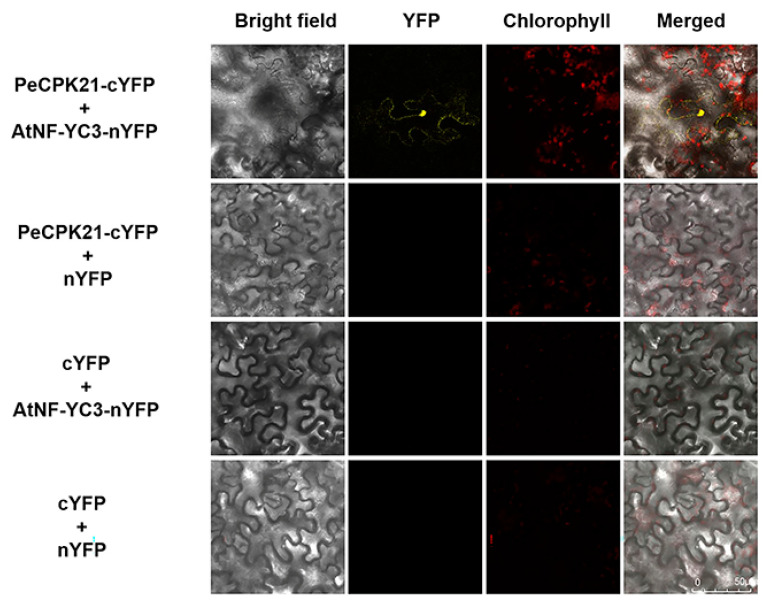
Bimolecular fluorescence complementation (BiFC) analysis between PeCPK21 and AtNF-YC3. *Agrobacterium tumefaciens* strains containing PeCPK21-cYFP or cYFP were mixed in an equal volume with the strains containing AtNF-YC3-nYFP or nYFP. The *Agrobacterium* suspensions were then injected into the abaxial surface of tobacco leaves (6 weeks old) using a needleless syringe. The leaves injected with *A. tumefaciens* were kept in the dark for 48–60 h. Tobacco leaves injected with the empty vector controls nYFP and cYFP served as negative controls. The red color is the chlorophyll autofluorescence. YFP: yellow fluorescent protein.

The interaction between PeCPK21 and AtNF-YC3 was further confirmed by BiFC assays in the leaves of *N. benthamiana*. The BiFC assays showed that co-expression of PeCPK21-cYFP and AtNF-YC3-nYFP in tobacco leaves resulted in yellow fluorescent protein (YFP) signaling in the nucleus and cytoplasm, which was not observed with other transforming combinations, such as PeCPK21-cYFP + nYFP, AtNF-YC3-nYFP + cYFP and cYFP + nYFP (Figure 5). The BiFC assays show that PeCPK21 was able to interact specifically with AtNF-YC3, and the protein–protein interaction probably occurred mainly in the nucleus and cytoplasm.

### 2.5. Cd Tolerance in AtNF-YC3-Transgenic Arabidopsis

**Figure 6:**
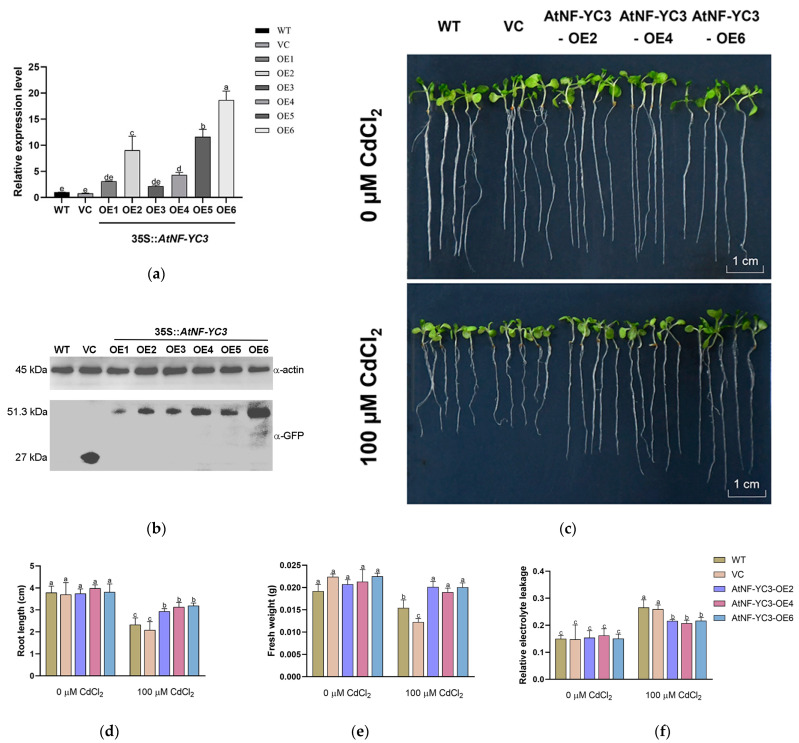
Cadmium tolerance testing of *AtNF-YC3* transgenic lines (OE2, OE4 and OE6). (**a**) RT-qPCR analysis of *AtNF-YC3*; (**b**) Western blotting of AtNF-YC3-GFP fusion protein in transgenic Arabidopsis; (**c**) representative images of phenotype tests under CdCl_2_ stress; (**d**) root length; (**e**) fresh weight; (**f**) relative electrolyte leakage. Seedlings of all tested lines, wildtype (WT), vector control (VC) and *AtNF-YC3*-OE2, OE4, and OE6 (T3 generation), were grown for 7 days in 1/2 Murashige and Skoog (MS) medium supplied with 0 or 100 μM CdCl_2_. Data in (**a**,**d**–**f**) are the mean of three individual plants, and bars with different letters indicate significant differences (*p* < 0.05).

To further determine whether the PeCPK21-interacting TF AtNF-YC3 could improve Cd tolerance, the A*tNF-YC3* gene was overexpressed in Arabidopsis. In this study, six transgenic Arabidopsis lines, OE1–OE6, were generated. Among the six transgenic lines, RT-qPCR showed that *AtNF-YC3* transcription was highest in OE6 and lowest in OE3 (Figure 6a). Western blotting confirmed that the AtNF-YC3-GFP protein was expressed in all transgenic lines, with protein abundance being highest in OE6 and lowest in OE1 (Figure 6b). Here, the transgenic lines OE2, OE4 and OE6 (T3 generation) were used for cadmium tests. The wildtype (WT), the vector control (VC) and three *AtNF-YC3*-OE lines were treated with CdCl_2_ (0 or 100 μM) for 7 days.

The growth of Arabidopsis seedlings was reduced by 100 μM CdCl_2_ (7 days, Figure 6c). It is noticeable that the root length of the three *AtNF-YC3*-OE lines under Cd stress was 26–53% higher than that of the WT and VC (Figure 6d). Similarly, the transgenic lines showed 23–64% greater fresh weight (per 15 plants) than the WT and VC (Figure 6e). Under the control conditions, no difference in root and plant growth was observed between the tested genotypes (Figure 6c–e). The CdCl_2_ treatment caused a significant increase in relative electrolyte leakage (EL) in all tested lines (Figure 6f). However, the EL in the *AtNF-YC3*-OE lines was 17–22% lower than in the WT and VC lines, indicating that membrane integrity was less affected by CdCl_2_ in the transgenic lines (Figure 6f). Collectively, AtNF-YC3 positively regulates Cd tolerance in Arabidopsis in terms of improved root length, fresh weight and membrane stability.

### 2.6. Root Cd Flux and Concentration

**Figure 7:**
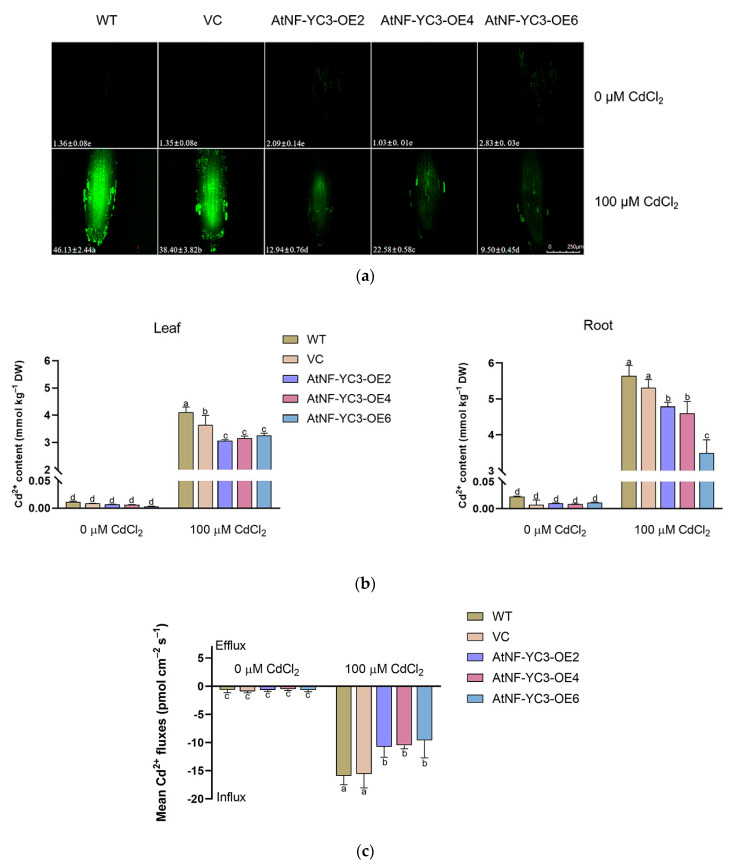
Root cadmium (Cd) concentration and flux in *AtNF-YC3* transgenic lines (OE2, OE4 and OE6). Seedlings of all tested lines, wildtype (WT), vector control (VC) and *AtNF-YC3*-OE2, OE4 and OE6 (T3 generation), were grown for 7 days in 1/2 Murashige and Skoog (MS) medium containing 0 or 100 μM CdCl_2_. (**a**) Cd concentration in the root cells. The green fluorescence of Leadmium™ Green AM was visualized with a confocal microscope (Leica SP8). (**b**) Cd content. Seedlings of WT, VC and *AtNF-YC3*-OE lines were watered with CdCl_2_ (0 or 100 μM) for one week. Leaves and roots were harvested, overdried and digested to measure the Cd content with an atomic absorption spectrometer. (**c**) Cd flux in the root tips. Net Cd flux was recorded continuously for 6–8 min at the apical zone. Each value (**a**) or column (**b**) is the mean of three to four individual plants, and different letters indicate significant differences (*p* < 0.05).

The Cd content in the root cells was detected with a fluorescent probe, Leadmium™ Green AM [5]. CdCl_2_ led to a marked increase in fluorescence intensity in the root cells, but the fluorescence in the *AtNF-YC3*-OE lines was only 16–45% of that in the WT and VC (Figure 7a). In contrast, the fluorescence in control plants was extremely low or undetectable (Figure 7a). The effect of AtNF-YC3 on Cd accumulation in the leaves and roots of Arabidopsis was also determined. The Cd content was measured using an atomic absorption spectrometer. The results showed that *AtNF-YC3* transgenic plants accumulated less Cd in leaves and roots than the WT and VC (Figure 7b). The steady-state Cd flux in the root tips was monitored using non-invasive micro-test technology (NMT). The Cd flux was almost undetectable in the roots of the control plants, while a remarkable influx was recorded in the roots exposed to 100 μM CdCl_2_ (Figure 7c). In particular, the transgenic *AtNF-YC3*-OE lines OE2, OE4 and OE6 showed a significantly lower Cd influx, and the flux rate was 58–67% of the values in the WT and VC plants (Figure 7c).

### 2.7. H_2_O_2_ Concentration, Activities and Transcription of Antioxidant Enzyme

**Figure 8:**
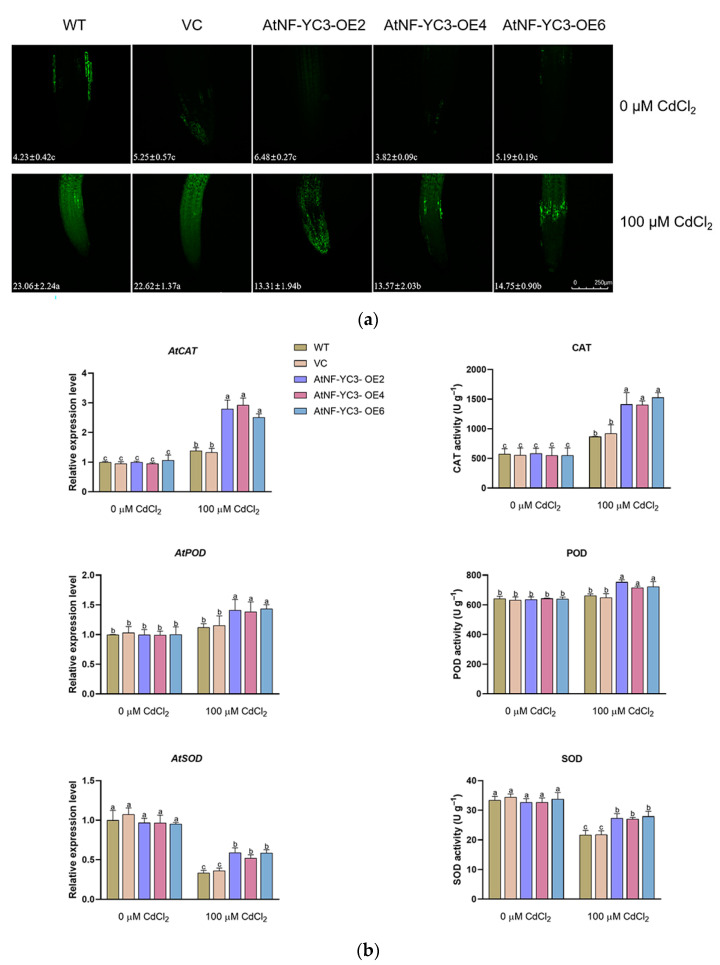
Hydrogen peroxide (H_2_O_2_) content, transcription and activity of antioxidant enzymes in *AtNF-YC3* transgenic lines. (**a**) H_2_O_2_ concentration in root cells. The green fluorescence of H2DCFDA was visualized with a confocal microscope (Leica SP8). Scale bar = 250 μm. (**b**) Transcription and activity of antioxidant enzymes. Seedlings of all tested lines, wildtype (WT), vector control (VC) and *AtNF-YC3*-OE2, OE4 and OE6 (T3 generation), were grown for 7 days in 1/2 Murashige and Skoog (MS) medium containing 0 or 100 μM CdCl_2_. Each value (**a**) or column (**b**) is the mean of three individual plants, and different letters indicate significant differences (*p* < 0.05).

Cadmium in general stimulates ROS accumulation in stressed plants [4,5,8]. The H_2_O_2_ content in root cells was detected with a fluorescent probe, H2DCFDA. CdCl_2_ exposure (100 μM) resulted in a significant rise in DCF fluorescence in root cells (Figure 8a). However, the *AtNF-YC3*-OE lines showed a significantly lower H_2_O_2_ level compared to the WT and VC. The control plants exhibited very low DCF fluorescence in all tested lines (Figure 8a).

The transcription levels of *AtCAT*, *AtPOD* and *AtSOD* as well as catalase (CAT), peroxidase (POD) and superoxide dismutase (SOD) activities were analyzed to determine the ability of Cd-treated plants to scavenge reactive oxygen species (ROS). CdCl_2_ exposure (100 μM) increased the transcription of *AtCAT* and *AtPOD* but inhibited the expression of *AtSOD* in all lines tested. Remarkably, the transcription of antioxidant enzymes was significantly higher in the *AtNF-YC3*-OE lines OE2, OE4 and OE6 regardless of the Cd up-regulation of *AtCAT* and *AtPOD* and Cd down-regulation of *AtSOD*. Compared to the WT and VC, the *AtNF-YC3* transgenic lines maintained higher activities of CAT, POD and SOD under Cd stress, which is consistent with the transcription of the coding genes (Figure 8b).

## 3. Discussion

### 3.1. PeCPK21 Interacts with AtNF-YC3 to Increase Cd Tolerance in Arabidopsis

We have previously shown that PeCPK21 increases Cd tolerance by interacting with various heavy metal stress-associated proteins (HMAPs) in transgenic Arabidopsis [4]. Here, we found that PeCPK21 also interacts with the transcription factor AtNF-YC3 by performing in vivo assays, including Y2H and BiFC (Figure 4 and Figure 5). Transcription of *AtNF-YC3* was strongly increased in transgenic seedlings when treated with CdCl_2_ (Figure 1), suggesting that AtNF-YC3 is responsible for Cd stress. Similarly, in rice, the *NFY-A6* gene is up-regulated by Cd treatment [41]. *AtNF-YC3* was overexpressed in Arabidopsis to determine whether the transcription factor interacting with PeCPK21 confers Cd tolerance. As shown in Figure 6, overexpression of *AtNF-YC3* enhanced Cd tolerance in terms of improved root length, fresh weight and membrane stability in Arabidopsis. Here, we confirmed for the first time that AtNF-YC3 improves Cd tolerance, although NF-YC transcription factors are required for plant response to ABA (e.g., NF-YC1 in *Physcomitrella patens* [29]; Cdt-NF-YC1 in bermudagrass [37]) and abiotic stress, such as salt (e.g., *NF-YC13* in indica rice [33]; *PgNF-YB02*, *PgNF-YC09* and *PgNF-YC07-04* in *Panax ginseng* [32]), drought (e.g., *NF-YC* in *Amaranthus hypochondriacus* [27]) and alkali stress (e.g., *NF-YC2* in *Medicago sativa* [36]). Our data show that PeCPK21 interacts with the transcription factor AtNF-YC3 to limit Cd uptake and enhance ROS degradation in transgenic plants.

### 3.2. PeCPK21 Interacts with AtNF-YC3 to Restrict Cd Uptake in Arabidopsis Roots

The confocal results showed that the *AtNF-YC3* transgenic lines effectively limited the buildup of Cd in Cd-exposed roots compared to the WT and VC (Figure 7). This resulted from the lower Cd influx into the root tips (Figure 7). We have shown that PeCPK21 interacts with heavy metal transport proteins and channels, PDF2.2, OPT3, COPT5 and annexin, to effectively limit Cd accumulation in the roots of *PeCPK21* transgenic lines [4]. Accordingly, we hypothesize that PeCPK21 also interacts with the transcription factor AtNF-YC3 to limit Cd uptake and thereby increase Cd tolerance. Therefore, PeCPK21 interacts with both the transcription factor AtNF-YC3 and heavy metal transport proteins to limit Cd uptake and accumulation under cadmium stress.

### 3.3. PeCPK21 Interacts with AtNF-YC3 to Improve Activities of Antioxidant Enzymes

Cd treatment resulted in lower H_2_O_2_ levels in *AtNF-YC3*-overexpressed plants compared to the WT and VC (Figure 8). The transcription of *AtPOD*, *AtCAT* and *AtSOD* was higher in the transgenic lines than in the WT and VC lines (Figure 8). In agreement with gene expression, the enzymatic activities were higher in the *AtNF-YC3*-overexpressed plants than in the WT and VC, regardless of Cd-stimulated CAT and POD and Cd-restricted SOD (Figure 8). The results suggest that AtNF-YC3 increases Cd tolerance by enhancing the activities of antioxidant enzymes in Arabidopsis. Similarly, the NF-YC transcription factor MsNF-YC2 positively regulates the activities of SOD and POD such that the increased antioxidant enzymes reduce the oxidative damage of H_2_O_2_ to the cell membrane in transgenic alfalfa [36]. Overexpression of garlic *AsNF-YC8* enabled tobacco plants to control ROS levels by activating antioxidant enzymes [24]. We have shown that PeCPK21 interacts with a variety of antioxidant enzymes, especially CDSP32, APX1, APX2, GPX3, PRXQ, TAPX and TRXM4, to maintain ROS homeostasis in *PeCPK21* transgenic lines under Cd stress [4]. Here, we hypothesize that PeCPK21 also interacts with AtNF-YC3 to scavenge the Cd-triggered ROS and increase cadmium tolerance. Therefore, PeCPK21 interacts directly with antioxidant enzymes or interacts with the transcription factor AtNF-YC3 to activate antioxidant enzymes under Cd stress.

## 4. Materials and Methods

### 4.1. Culture of Plant Materials

*Arabidopsis thaliana* wild type (WT), vector control (VC) and *PeCPK21*-tansgenic lines OE3, OE7 and OE10 were surface sterilized, germinated and grown in 1/2 Murashige and Skoog (MS) medium (0.8% agar and 1% sucrose, *w/v*) containing 0 or 100 μM CdCl_2_. The seedlings were used for quantitative real-time PCR analyses of *AtNF-YC3* expression [4]. The primers used for RT-qPCR are shown in Supplementary Table S1.

### 4.2. AtNF-YC3 Cloning and Bioinformatic Analysis

Total RNA was isolated from Arabidopsis using Trizol reagent (Invitrogen, Carlsbad, CA, USA). The reverse transcriptase kit HiFiScript RT MasterMix (Cowin Bio, Taizhou, China) was used for first-strand cDNA synthesis. *AtNF-YC3* was cloned by PCR amplification and the 50 μL reaction mixture contained the cDNA product (2 μL), forward and reverse primers (10 μM, 1 μL) and KOD OneTM PCR Master Mix (TOYOBO, Osaka, Japan, 25 μL). The primer sequences for gene cloning are listed in Supplementary Table S2. The PCR product was gel purified and sequenced for multiple sequence alignments and phylogenetic analyses [42]. The GenBank accession numbers of the NF-YC proteins are shown in Supplementary Table S3.

### 4.3. Subcellular Localization of PeCPK21 and AtNF-YC3

For the subcellular localization test, the *PeCPK21* and *AtNF-YC3* sequences were inserted into the pCAMBIA-1300 GFP vector and the pBI121-mCherry vector, respectively. The recombinant plasmid pCAMBIA-1300 GFP-*PeCPK21* and pBI121-mCherry-*AtNF-YC3* was then transformed into *A. tumefaciens* (strain GV3101) and subsequently co-infiltrated into tobacco leaves. The fluorescence of mCherry and GFP was analyzed using a Leica confocal microscope (TCS SP8, Leica Microsystem GmbH, Wetzlar, Germany).

### 4.4. Yeast Two Hybrid

The Matchmaker Gal4-based Yeast two-hybrid system (Clontech Laboratories, Inc. Mountain View, CA 94043, USA) was used to verify the interaction between PeCPK21 and AtNF-YC3. The coding sequences (CDSs) of *AtNF-YC3* and *PeCPK21* were ligated to pGADT7 and pGBKT7 vectors, respectively. The recombinant plasmids were co-transformed into yeast strain AH109, which was then cultured on synthetic dropout (SD)/-Leu/-Trp medium and SD/-Leu/-Trp/-His/-Ade medium to test the possible protein–protein interactions. The primers used for the yeast two hybrid (Y2H) assay are shown in Supplementary Table S2.

### 4.5. Bimolecular Fluorescence Complementation

BiFC assays were performed to determine whether PeCPK21 interacts with AtNF-YC3 in planta. The CDSs of *PeCPK21* and *AtNF-YC3* were ligated to pSPY-CE and pSPY-NE vectors, respectively. Plasmids containing *PeCPK21*-YCE and *AtNF-YC3*-YNE were transferred into *A. tumefaciens* strain GV3101 (pSoup19 GV3101). The transgenic strains containing *PeCPK21*-YCE/YCE were thoroughly mixed with *AtNF-YC3*-YNE/YNE at a volume ratio of 1:1 and then maintained at 28 °C for 2–4 h. The *A. tumefaciens* strains were infiltrated into tobacco leaves and kept in the dark for 48 h. Yellow fluorescent protein (YFP) fluorescence was finally detected using a confocal microscope (TCS SP8, Leica Microsystem GmbH, Wetzlar, Germany). Supplementary Table S2 lists the primers used for the BiFC assays.

### 4.6. Overexpression of AtNF-YC3 in Arabidopsis

The CDS of *AtNF-YC3* was cloned into the pCAMBIA-1300-GFP vector with the *KpnI* and *SalI* sites and driven by the CaMV35S promoter. The constructed *AtNF-YC3-GFP* was transformed into *A. tumefaciens* (strain GV3101), which was used for plant transformation [4]. The hygromycin (25 mg/L)-resistant plants (T1 generation) were selected and used to produce the homozygous transgenic lines of the T2 and T3 generations. Six transgenic lines overexpressing *AtNF-YC3*, i.e., OE1, OE2, OE3, OE4, OE5 and OE6, were obtained and verified by RT-qPCR and Western blotting.

### 4.7. Real-Time Quantitative PCR Analysis

Total RNA was extracted from WT Arabidopsis, the VC, *PeCPK21* transgenic lines (OE3, OE7 and OE10) and *AtNF-YC3* transgenic lines OE2, OE4 and OE6 following the previously described method. The RNA samples were purified, quantified and utilized for RT-qPCR analysis with a LineGene 9600 Plus Real-Time Quantitative PCR System (FQD-96A, BIOER Technology, Hangzhou, China). *AtACT2* served as an internal reference gene for Arabidopsis [42]. The transcription of *AtNF-YC3*, *AtCAT*, *AtPOD* and *AtSOD* in Arabidopsis was assessed in both control and Cd-stressed plants. The specific primers for the target and reference genes can be found in Supplementary Table S1.

### 4.8. Extraction of Total Protein from Arabidopsis and Western Blotting

The leaves of 4-week-old *Arabidopsis thaliana* were ground in liquid nitrogen and the appropriate protein extract was added. The mixture was shaken for 1 min and then placed on ice for 10 min. The samples were centrifuged at 13,000 rpm (4 °C) for 15 min and the supernatant (total protein) was used for Western blotting. The supernatant was mixed with 5 × SDS loading buffer, completely denatured at 95 °C for 5 min, then cooled down on ice. The mixture was subjected to SDS-PAGE at 120 V for 2 h and transferred to polyvinylidene fluoride (PVDF) membranes. The immunoblots were probed with anti-GFP antibodies, and equal loading was confirmed by probing with anti-Actin antibodies (ABclonal Technology, Wuhan, China) [43].

### 4.9. Phenotype Test under Cd Stress

Seeds of wild-type Arabidopsis (Col-0), the vector control (VC) and the *AtNF-YC3*-overexpressing lines OE2, OE4 and OE6 were surface-sterilized and germinated in 1/2 MS solid medium (0.8% agar, 1% sucrose, *w/v*) containing 0 or 100 μM CdCl_2_. After vernalization at 4 °C for 48 h, the seeds were germinated and grown at 22 °C with an illumination of 60 μmol m^−2^ s^−1^. Fresh weight of plants, root length, and electrolyte leakage (EL) were examined after 7 days of CdCl_2_ treatment [42].

### 4.10. Cellular Cd and H_2_O_2_ Measurement

Cd and H_2_O_2_ concentrations in root cells were measured using the Leadmium^TM^ Green AM fluorescent probe (Invitrogen, Carlsbad, CA, USA) and H2DCFDA (Molecular Probes, Biorigin, Beijing, China) as previously described [5,42]. Briefly, the WT, VC and *AtNF-YC3*-overexpressing lines (OE2, OE4 and OE6) treated with or without CdCl_2_ (100 μM, 7 d) were incubated with 50 μM Leadmium^TM^ Green AM for 1 h or with 10 μM H2DCFDA for 0.5 h in the dark. The roots were then sampled to measure the fluorescence intensities of Cd and H_2_O_2_ using a Leica confocal microscope (TCS SP8, Leica Microsystem GmbH, Wetzlar, Germany).

### 4.11. Recordings of the Cd flux in the Roots

Net Cd fluxes in root tips were recorded with microelectrodes equipped with the non-invasive micro-test system (NMT-YG-100, Younger USA, LLC, Amherst, MA, USA) [5,6,44]. Roots were collected from control and CdCl_2_-treated plants of WT, VC and *AtNF-YC3*-overexpressing lines (OE2, OE4 and OE6) and immediately equilibrated in measuring solutions for 30 min. For each treatment, five individual plants were used for flux recording. Cd flux rates were calculated using the program JCal version V3.2.1 (http://www.xuyue.net/, accessed on 18 July 2023).

### 4.12. Cd Enrichment in Arabidopsis

The WT, VC and *AtNF-YC3*-OE lines were raised in a culture room at 25 ± 1 °C with a photoperiod of 16 h light/8 h dark, a light intensity of 50 μmol m^−2^ s^−1^ and a relative humidity of 50–60%. The potted seedlings were watered with 0 or 100 µM CdCl_2_ for 7 days. Then, the seedlings of all tested genotypes were harvested and dried for 70–80 °C over 3 d. The dried samples were digested in 2.5 mL concentrated HNO_3_ and 0.5 mL HClO_4_ using a microwave acceleration system (Titan MPS Microwave Sample Preparation System, Perkin-Elmer, Waltham, MA, USA). Cd concentrations were determined using the PerkinElmer PinAAcle900T spectrometer (Perkinelmer, Waltham, MA, USA) [44]. Three biological samples were used for each treatment and ion analysis was repeated at least three times.

### 4.13. Measurement of the Activities of the Antioxidant Enzymes

The WT, VC and *AtNF-YC3*-overexpressing lines (OE2, OE4 and OE6) were exposed to 0 or 100 μM CdCl_2_ for 7 days. The control and stressed plants were collected to measure the activities of antioxidant enzymes using the assay kits for SOD, POD and CAT (Njjcbio, Nanjing, China) [45].

### 4.14. Statistical Analysis

All experimental data were statistically analyzed using SPSS version 19.0 (IBM Corporation, Armonk, NY, USA). Significant differences between mean values were determined using the Duncan Multiple Range Test (DMRT). For post hoc multiple comparisons, the least significant difference (LSD) method was used. A value of *p* < 0.05 was considered significant unless otherwise stated.

## 5. Conclusions

We conclude that PeCPK21 interacts with the transcription factor AtNF-YC3 to reduce Cd accumulation and strengthen the antioxidant system to reduce ROS triggered by Cd stress. This enables the transgenic plants to adapt to the Cd environment. This study highlights the regulatory role of PeCPK21 and AtNF-YC3 in Cd stress tolerance, which can be utilized to improve Cd tolerance in higher plants.

## Data Availability

The raw data supporting the conclusions of this article will be made available by the authors on request.

## Supplementary Materials

The following supporting information can be downloaded at https://www.mdpi.com/article/10.3390/ijms25137214/s1.

## Abbreviations

ABA: abscisic acid; *ACT2*: *Actin2*; APX: ascorbate peroxidase; APX1: ascorbate peroxidase 1; APX2: ascorbate peroxidase 2; AVA-P2: V-type proton ATPase proteolipid subunit; BiFC: bimolecular fluorescence complementation; CAT: catalase; CDS: coding sequence; Cd: cadmium; CDSP32: chloroplastic drought-induced stress protein of 32 kDa; COPT5: copper transporter 5; CPK: calcium-dependent protein kinase; DMSO: dimethyl sulfoxide; EL: relative electrolyte leakage; GPX3: glutathione peroxidase; HAP: heme-activated protein; HMAPs: heavy metal stress-associated proteins; NF-Y: nuclear transcription factor Y; NMT: non-invasive micro-test technology; OPT3: oligopeptide transporter 3; PDF2.2: plant defensin-like protein 2.2; PIP1–1: plasma membrane intrinsic protein 1-1; PIP2A: plasma membrane intrinsic protein 2A; PIP2–7: plasma membrane intrinsic protein 2-7; POD: peroxidase; PRXQ: thioredoxin superfamily protein; ROS: reactive oxygen species; RT-qPCR: quantitative real-time polymerase chain reaction; SD: synthetic dropout; SOD: superoxide dismutase; TAPX: thylakoid ascorbate peroxidase; TF: transcription factor; TRXM4: thioredoxin M4; VC: vector control; VHA-B1: V-type proton ATPase subunit B1; VHA-C: V-type proton ATPase subunit C; WT: wild type; XTH: xyloglucan endotransglucosylase/hydrolase; Y2H: yeast two hybrid.
