# Supplementary material for: Populus euphratica CPK21 Interacts with NF-YC3 to Enhance Cadmium Tolerance in Arabidopsis
Source: Int J Mol Sci. 2024 Jun 29;25(13):7214. doi: 10.3390/ijms25137214 (PMC11240976; doi:10.3390/ijms25137214)
Supplement: Supplementary file 1 [file ijms-25-07214-s001.zip › ijms-3082112-supplementary.pdf]

International Journal of
Molecular Sciences

MDPI

Supplementary Table S1. Primers used for quantitative real-time PCR.

Gene	Forward primer	Reverse prime
AtACTIN2	GGTAACATTGTGCTCAGTGGTGG	AACGACCTTAATCTTCATGCTGC
AtCAT	AATATGCTGACGATGAGGATGC	CAAGAATCAAGGAGGTAGGAGATG
AtNF-YC3	AGCTTACGTTAAGGTCTTGG	CGGGTTACCCATAACCATTC
AtPOD	CGTGCCCTTCATATTGTTGG	GACGCCATCAACAACGAGTC
AtSOD	AGGAAACATCACTGTTGGAGAT	GAGTTTGGTCCAGTAGAGGGAA

Supplementary Table S2. Primers used for gene cloning.

Gene	primers
AtNF-YC3- pBI121	5′-CACGGGGGACTCTAGAATGGATCAACAAGGACAATCATCAG-3′<br>5′-GACCACCCGGGGATCCATTGTCAGGATCCTGCTGCTCAG-3′
AtNF-YC3- pCAMBIA-1300 GFP	5′-GGGGCCCGGGGTCGACATGGATCAACAAGGACAATCATCAG-3′<br>5′-CCCTTGCTCACCATGGTACCATTGTCAGGATCCTGCTGCTCAG-3′
AtNF-YC3- pGADT7	5′-GGAGGCCAGTGAATTCATGGATCAACAAGGACAATCATCAG-3′<br>5′-CGAGCTCGATGGATCCCTAATTGTCAGGATCCTGCTGC-3′
AtNF-YC3- pMD18T	5′-ATGGATCAACAAGGACAATCATCAG-3′<br>5′-CTAATTGTCAGGATCCTGCTGCTCAG-3′
AtNF-YC3- YNE	5′-CGCCACTAGTGGATCCATGGATCAACAAGGACAATCATCAG-3′<br>5′-TCCATCCCGGGAGCGGTACCATTGTCAGGATCCTGCTGCTCAG-3′
PeCPK21- pCAMBIA-1300 GFP	5′-GGGGCCCGGGGTCGACATGGGTTGTTTTAGTAGCAAAGA-3′<br>5′-CCCTTGCTCACCATGGTACCATAGAGGCTCGGTGCAGG-3′
PeCPK21- pGBKT7	5′-CATGGAGGCCGAATTCATGGGTTGTTTTAGTAGCAAAGA-3′<br>5′-GCAGGTCGACGGATCCTTAATAGAGGCTCGGTGCAGG-3′
PeCPK21- YCE	5′-CGCCACTAGTGGATCCATGGGTTGTTTTAGTAGCAAAGA-3′<br>5′-TACATCCCGGGAGCGGTACCATAGAGGCTCGGTGCAGG-3′

Supplementary Table S3. Accession numbers of NF-Y orthologs.

	**Accession number**
AtNF-YC1	NP_190428.1
AtNF-YC2	NP_001077726.1
AtNF-YC3	NP_175880.1
AtNF-YC4	NP_001032130.1
AtNF-YC5	NP_199860.1
AtNF-YC6	NP_199859.1
AtNF-YC7	NP_199858.1
AtNF-YC8	NP_198143.1
AtNF-YC9	NP_172371.1
AtNF-YC11	NP_187854.2
AtNF-YC12	NP_198630.2
NtNF-YC1	XP_016511061.1
NtNF-YC9	XP_016515372.1
OsNF-YC2	XP_015628319.1
OsNF-YC4	XP_015627520.1
OsNF-YC6	XP_015611933.1
PtHAP5.3	UER43237.1
PtNF-YC1	UER43240.1
PtrNF-YC1	XP_024442550.1
PtrNF-YC2	XP_002310081.2
PtrNF-YC3	XP_002319553.1
PtrNF-YC4	XP_024438371.2
PtrNF-YC9	XP_052311030.1
ZmNF-YC1	NP_001147992.1
ZmNF-YC2	AQK81605.1
ZmNF-YC4	NP_001136950.1
ZmNF-YC6	NP_001149301.1
